# New Insights into the Roles of p53 in Central Nervous System Diseases

**DOI:** 10.1093/ijnp/pyad030

**Published:** 2023-06-20

**Authors:** Haili Li, Ze Zhang, Huixin Li, Xinyu Pan, Yue Wang

**Affiliations:** Department of Neurosurgery, the First Affiliated Hospital of Shandong First Medical University and Shandong Provincial Qianfoshan Hospital, Jinan, China; School of Clinical and Basic Medical Sciences, Shandong First Medical University and Shandong Academy of Medical Sciences, Jinan, China; Department of Neurosurgery, the First Affiliated Hospital of Shandong First Medical University and Shandong Provincial Qianfoshan Hospital, Jinan, China; School of Clinical and Basic Medical Sciences, Shandong First Medical University and Shandong Academy of Medical Sciences, Jinan, China; School of Clinical and Basic Medical Sciences, Shandong First Medical University and Shandong Academy of Medical Sciences, Jinan, China; School of Clinical and Basic Medical Sciences, Shandong First Medical University and Shandong Academy of Medical Sciences, Jinan, China; Department of Neurosurgery, the First Affiliated Hospital of Shandong First Medical University and Shandong Provincial Qianfoshan Hospital, Jinan, China; School of Clinical and Basic Medical Sciences, Shandong First Medical University and Shandong Academy of Medical Sciences, Jinan, China; Medical Science and Technology Innovation Center, Shandong First Medical University and Shandong Academy of Medical Sciences, Jinan, China

**Keywords:** Neurological diseases, p53 mutation, brain tumors, Alzheimer disease, signaling pathways

## Abstract

The transcription factor p53, a widely accepted tumor suppressor, regulates the expression of many oncogenes and their downstream signaling pathways, resulting in a series of biological outcomes. Mutations and deletions of the *p53* gene often occur in tumor tissues and are involved in their development. In addition to its role in tumors, p53 has a widespread expression in the brain and participates in most cell processes, such as dendrite formation, oxidative stress, apoptosis, autophagy, DNA repair, and cell cycle arrest. Therefore, abnormalities in p53 and its related signaling pathways play an important role in the diagnosis and treatment of central nervous system diseases. This review mainly discusses the latest findings regarding the role of p53 in some central nervous system diseases, such as brain tumors, Alzheimer disease, Parkinson disease, autism, epilepsy, spinocerebellar ataxia, and so on, to provide a comprehensive interpretation of the treatment of neurological diseases from a new perspective.

## INTRODUCTION

The *p53* gene, located on the short arm of chromosome 17, was discovered over 40 years ago, and since then its functions have been investigated by an increasing number of scientists (Levine, 2020). The p53 protein is widely distributed in the body, and there is a less significant difference in the expression of p53 protein during body development. The p53 protein consists of 3 core functional domains: the N-terminal domain that plays a role in transcription activation, the C-terminal domain that plays a regulatory role, and the DNA-binding domain, which specifically binds to the promoters of genes (Kato et al., 2003). As an important transcription factor, p53 regulates a variety of genes, forming a delicate and complex regulatory network to maintain cell homeostasis and physiological processes. The key factors involved in the cell cycle checkpoint are regulated by p53, such as 14-3-3δ and GADD45; p53 also regulates cell apoptosis through Bax/Bcl-2 and Fas/Apol to maintain genome stability (Sun, 2006). Moreover, the posttranslational activity of the p53 protein is regulated by multiple modification levels, such as ubiquitination, phosphorylation, and acetylation (Liu et al., 2019). In nonstressed situations, the level of p53 expression is very low, and its function is inhibited by the MDM2 ubiquitin/protease degradation pathway; thus, MDM2 has an antagonistic effect on p53 (Wu and Prives, 2018). The transcription of some genes, such as PUMA, PARP, and Pml involved in apoptosis and senescence, can be activated by phosphorylated p53 (Wiman, 2013). However, when cells are stimulated by stress signals, such as oxidation stress, hypoxia, oncogene activation, DNA double-strand breaks, or telomere damage, the p53 protein is rapidly activated to maintain the integrity of the cell genome by inducing apoptosis, cell cycle arrest, and senescence (Yoshida and Miki, 2010). Therefore, p53 is often called the guardian of the cell genome.

Because p53 is widely distributed in brain tissue, it inevitably participates in the regulation of various neural functions, such as development, axon regeneration, and neurite outgrowth. During the neuronal differentiation of PC12 cells induced by nerve growth factor, some specific differentiation-related target genes are activated by p53 proteins, such as tfcp2l4/grhl3 and Wnt7b. Among them, tfcp2l4/grhl3 is involved in the development of individual embryo ectoderm, and Wnt7b is involved in the growth of neuronal dendrites (Brynczka et al., 2007). Coronin1b and GTPase Rab13, p53 transcriptional targets, can both regulate neurite growth, and the function of GTPase Rab13 is mainly related to the cytoskeleton. Acetylation of p53 has been reported to promote neurite growth by modulating the high expression of Coronin1b and GTPase Rab13 (Di Giovanni et al., 2006). GAP-43 is an axon growth-related protein, whose function is to promote the growth of neuronal axons. p53 acetylation can also bind to the GAP-43 promoter and drive axon growth (Tedeschi et al., 2009). Similar to the regulation of neurite growth, p53 also plays an important role in the regeneration of neuronal axons in vivo. Overexpression of p53 in sensory neurons and retinal ganglion can significantly promote regeneration of sensory axons and optic nerves (Ma et al., 2019). In addition, p53-related signaling pathways are also involved in some processes of nerve injury, such as spinal cord nerve injury caused by certain diseases or brachial plexus injury caused by trauma (Ma et al., 2017; Suroto et al., 2021).

**Figure 1.**
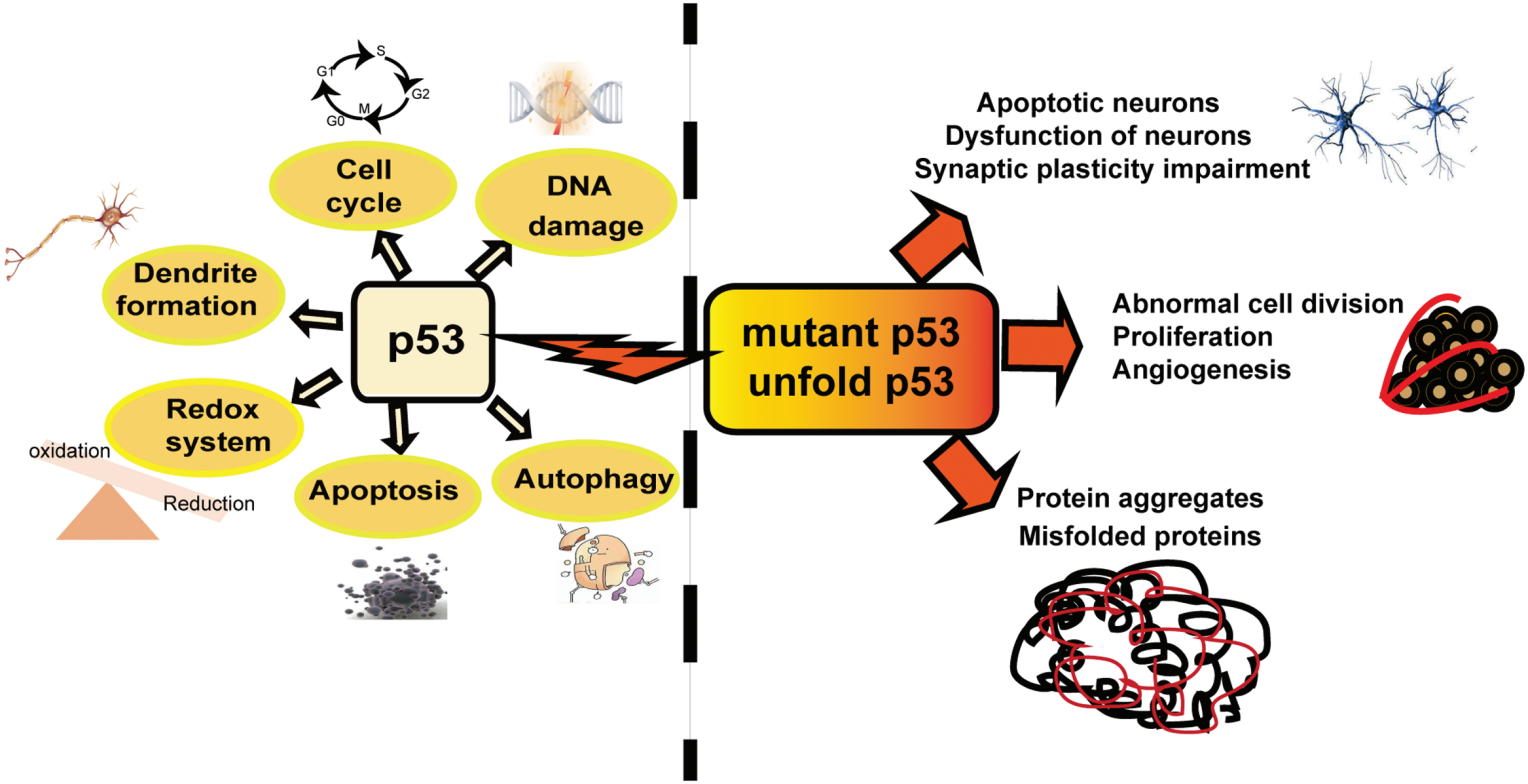
The regulation mechanism of p53 on the nervous system.

In summary, p53 is involved in regulating multiple signaling pathways that maintain the physiological homeostasis of nerve cells (**Figure 1**). Thus, abnormal expression of p53 protein can lead to the occurrence and development of neurological diseases. p53 is an important cancer suppressor factor that has a high mutation frequency in human cancers. Through the pathological analysis of a large number of tumor patients, approximately 50% of the tumor samples had p53 deletion or mutation, including brain tumors (Tokino and Nakamura, 2000). According to The Cancer Genome Atlas database, 3 hotspots (p53R273, p53R175, and p53R248) have the highest mutation frequency in glioma (Uno et al., 2005). In addition, the involvement of p53 in some central nervous system (CNS) diseases such as some neurodegenerative diseases (Alzheimer disease and Parkinson disease), autism, spinocerebellar ataxia, and other neurological diseases have been reported, thus providing a comprehensive interpretation of the diagnosis and treatment of neurological diseases from a new perspective.

## P53 AND BRAIN TUMORS

Brain tumors derived from glial cells are one of the top 10 most common malignant cancers. Gliomas represent nearly 80% of all malignant tumors in the brain and are named according to different glial cell types (Ohgaki and Kleihues, 2007). Among all brain tumors, diffuse glioma is the most clinically occurring malignant brain tumor, among which glioblastoma (GBM), as defined by the World Health Organization, has the worst prognosis in adults. However, in children, medulloblastoma is the malignant brain tumor with the highest incidence (Polkinghorn and Tarbell, 2007; Louis et al., 2021). Brain tumors can be divided into 2 categories: primary and secondary GBM. Clinical studies have suggested that p53 exhibits different mutation frequencies in these 2 categories (Yang et al., 2018). In primary GBM, p53 mutations occur less frequently in approximately 28% of cases and are spread throughout the gene, but in secondary GBM, p53 mutations are found in approximately 60% (Ohgaki and Kleihues, 2007). If alteration of p53 pathways is taken into consideration, the number of mutations is even higher in GBM. Approximately 90% of GBM cell lines have concomitant dysregulation of the ARF-MDM2-p53 signaling pathway. It is well known that patients with Li-Fraumeni syndrome have mutations in the p53 germline. However, most patients with this disease have accompanying brain tumors (Malbari and Lindsay, 2020). It has been hypothesized that genomic instability in these tumors may result from p53 mutation. Transgenic mouse models with p53 deficiency or mutation have an increased incidence of medulloblastoma. Studies have shown that p53 influences cell cycle arrest through regulation of the cell cycle–related factor PARP or the cell cycle regulatory protein Rb (Marino et al., 2000; Tong et al., 2003).

According to numerous previous reports, there are 2 potential mechanisms for the p53 mutation in cancer development, one leading to a loss of function. For example, *p21*^*(WAF1/CIP1)*^ is one of the target genes for p53. A mutation in P53V143A can block the transcription of p21 and its function of inducing cell cycle arrest, leading to cell cycle disorder and promoting the occurrence of malignant glioma (Bartussek et al., 1999). In addition, p53 can regulate the transcription of some microRNAs. p53 can activate the expression of miR-34a, which can also promote the expression of p53 by inhibiting the expression of the downstream target gene MAGE-A that can bind with MDM2 to repress p53 expression. Thus, p53 and miR-34a form a positive feedback loop, which can be widespread in the case of mutant p53, leading to the occurrence of glioma through loss of function (Weeraratne et al., 2011; Zhang et al., 2019). The other mechanism is termed gain of function, which was first observed in p53-null cells, while mutant p53 (pM8) cells formed lethal tumors in mice (Wolf et al., 1985; Stein et al., 2019). The inflammatory response induced by p53 mutation gain of function promotes the worsening outcome of glioma patients. By transcriptome sequencing of clinical glioma samples of p53 mutants (19NS, 84NS, 157NS, and 528NS), the results showed that p53 mutants could promote the upregulation of inflammatory factor CCL2 and chemotactic gene tumor necrosis factor-α expression in glioma (Ham et al., 2019). In GBM, the p53R273H mutation is able to form complexes with factors such as CBP, which can promote histone acetylation, and NFY, which promotes the transcription of mutated p53, thus promoting cancer cell proliferation, invasion, and survival (Huang et al., 2013). p53R175H, one of the hotspot mutants of p53, promotes high expression of miR-128 and its host genes by binding to ARPP21 (Donzelli et al., 2012). However, wild-type p53 does not function to regulate miR-128 transcription. In short, mutants of p53 protein are usually expressed in large quantities in tumors, showing carcinogenic function, but their specific mechanisms still need to be further studied and confirmed.

Even though current treatment with surgical resection, chemotherapy, and radiation is effective in brain tumors, many side effects and worse prognosis (such as reduced intellect) often occur (Omuro and DeAngelis, 2013). Therefore, it is very important to choose the appropriate treatment for different individuals and avoid unsuitable excessive therapies. It was recently reported that p53 might be a specific molecular marker for predicting the sensitivity of chemotherapeutic drugs during brain tumor treatment (Kristensen et al., 2019). Analyzing the postmortem sample tissue of GBM patients, patients who received TMZ treatment for more than half a year also had p53 overexpression, implying that p53 expression could predict the outcomes of patients receiving TMZ chemotherapy for a long time (Malkoun et al., 2012). Of note, p53 mutation is also associated with chemoresistance and poor prognosis of some cancers. For example, the p53R175H mutant inhibited tumor cell apoptosis in lung cancer and increased tumor resistance to the chemotherapy drugs cisplatin and doxorubicin (Chiang et al., 2021). In addition to inducing tumor cell resistance to chemotherapy, p53 mutants can also resist the sensitivity of cancer cells to radiation therapy, such as the p53V143A mutation (Li et al., 2019b). However, the relationship between p53 mutations and drug resistance in brain tumors has not been clarified thus far and is worth exploring in the future. Because these complex drug resistance outcomes in cancer cells after chemotherapy or radiotherapy depend on the type of p53 mutation, the treatment of p53 with certain small-molecule drugs or peptides, and then degradation or restoration of wild-type mutant p53 activity, may contribute to increased sensitivity in the treatment of tumors containing mutant p53 (Zhou et al., 2019).

## P53 AND ALZHEIMER DISEASE (AD)

AD is a common neurodegenerative disease, and its neuropathological features are the accumulation of amyloid-β (Aβ) and nerve fiber tangles formed by tau hyperphosphorylation, resulting in neuronal dysfunction (De-Paula et al., 2012; Scheltens et al., 2021). A high level of cell death has been observed in the brains of AD patients, which is because of the aggregation of tau and Aβ proteins forming nerve fiber tangles, making it a complex disease. The p53 protein has been reported to be involved in the regulation of neuronal death in the brains of patients with mild cognitive impairment or AD (Lanni et al., 2012; Salech et al., 2022). Some researchers have found that p53 is highly expressed in the inferior parietal lobe of the cerebral cortex in AD patients (Cenini et al., 2008). In the brains of patients aged 65 years and older, a large number of Aβ diffuse plaques were scattered in the cerebellar cortex, and high expression of p53 protein was also detected in cerebellar neurons (Maślińska et al., 2017). High expression of p53 protein was also found in transgenic mouse models carrying the Abeta42 mutation, which plays an important role in the pathogenesis of AD (Ohyagi et al., 2005). Both AD and mild cognitive impairment are associated with elevated oxidative damage in the brain, and p53 can protect cell survival by activating some antioxidant factors or prompting the process of neuronal apoptosis, such as manganese superoxide dismutase (MnSOD) and TIGAR. MnSOD is associated with mitochondrial dysfunction, and TIGAR is a p53-induced regulator of glycolysis and apoptosis (Sablina et al., 2005; Budanov, 2014). However, in AD mouse models with APP and PS1 mutations, primary neurons showed decreased MnSOD expression, which is induced by p53 (Sompol et al., 2008). This finding supported that impaired p53 activity participates in the pathogenesis of AD through dysregulation of antioxidant genes (Abate et al., 2020). As mentioned before, p53 is involved in Aβ deposition in normal senescent patients by activating proapoptotic p53 target genes such as CDK5 (Lee et al., 2007). Neuronal death in AD is not only accompanied by a high level of p53 gene expression but also functions to bind other pathways and genes to induce the accumulation of Aβ and accelerate the occurrence of AD (Zia et al., 2021). The mTOR pathway plays a key role in the development of AD by regulating protein synthesis and degradation, including promoting the phosphorylation of p53 and tau (Yates et al., 2013; Perluigi et al., 2021). The Wnt signaling pathway is another important pathway that cooperates with p53 to participate in the pathogenesis of AD (Zabłocka et al., 2021). In addition, the interaction of glycogen synthase kinase-3β (GSK-3β) with p53 promotes tau phosphorylation and increases plaque and tangle levels (Proctor and Gray, 2010). Therefore, molecules such as GSK-3β, p-GSK-3β, and p53, which can be detected in peripheral blood lymphocytes of AD patients, may be potential therapeutic targets in AD (Arafa and Elghazawy, 2017; Kumari et al., 2022).

As mentioned above, p53 is present at a low level in normal cells. In addition to posttranslational modifications, the conformational state of p53 also affects intracellular stability. Unfolded p53 has been detected in early-onset AD patients (Buizza et al., 2012). Unfolded p53 can specifically alter p53 DNA binding properties. When DNA damage occurs, the p53 protein induces G1 phase arrest of the cell cycle to aid genomic repair, but this arrest disappears when unfolded p53 is detected during AD progression (Zhang et al., 2021). Therefore, the conformationally altered p53 in AD is likely to contribute to G1/S checkpoint dysfunction and increase the rate of brain atrophy during the development of AD (Zhou and Jia, 2010). Unfolded p53 may not only be a high-risk factor for the progression of AD patients, but it may have the same role in other CNS diseases (Lanni et al., 2010; Abate et al., 2020; García et al., 2021). In addition, the presence of p53 isoforms leads to the occurrence of AD. The p53 protein isoform Δ40p53 in mice can also accelerate the aggregation of tau protein and further lead to synaptic defects and cognitive decline (Abate et al., 2020). p53 can also promote the expression of microRNAs, such as miR-34c, which participates in the pathological process of AD by regulating its binding target molecules. MiR-34c and p53 were both significantly elevated in blood samples from AD patients, along with activation of the ROS/JNK pathway and a reduction in the synaptotagmin1 (Syt1) protein (Shi et al., 2020). Tau mRNA 3’-untranslated regions can be regulated by miR-34, which can initiate transcription through p53. Thus, p53 also regulates the expression of tau through miRNAs (Dickson et al., 2013; Rokavec et al., 2014).

Targeting p53 as a therapeutic target to prevent or treat AD remains a great challenge. Although p53 is related to neuronal death and is involved in the pathological processes of AD, only in vitro evidence has been promising, and in vivo studies are needed to support the potential for p53 targeting to be translated into the clinic. As mentioned above, phospho-p53 (Ser15) and phospho-p21 (thr145) are biomarkers for the diagnosis of AD in peripheral blood lymphocytes of patients with AD (Tan et al., 2012). In addition, p53 is involved in many intracellular pathways, such as oxidative stress-induced apoptosis. Unfortunately, the activation of these signals has not been adequately demonstrated in neurons. Resveratrol is a natural phytoestrogen with neuroprotective properties due to its antioxidant properties (Lange and Li, 2018). Because p53 regulates oxidative stress–induced apoptosis, resveratrol can also effectively suppress the apoptotic activities of both p53 and FOXO, conferring neuronal protection in AD (Gomes et al., 2018). Therefore, we should pay more attention to the changes in intracellular pathways impacted by p53 to target changes in multiple factors and delay the development and deterioration of AD.

## P53 AND PARKINSON DISEASE (PD)

PD is another common clinical neurodegenerative disease in elderly individuals that mainly manifests as α-synuclein aggregation in dopaminergic neurons in the midbrain substantia nigra (Hayes, 2019). As mentioned above, p53 is involved in the process of neuronal oxidative stress, apoptosis, and abnormal protein aggregation, and these mechanisms are also involved in regulating the occurrence and progression of PD (Luo et al., 2022). In a PD cell model, the expression level of p53 protein was very high (Li et al., 2016). In PD patients and animal models of disease, p53 overexpression in the substantia nigra region of the brain has a direct regulatory relationship with dopaminergic neuron death (Mogi et al., 2007; Sekar and Taghibiglou, 2020). Therefore, a number of neuronal cell death pathways are activated in PD, such as neuronal apoptosis, oxidative damage, and abnormal protein aggregation (Luo et al., 2022). *DJ-1* is a PD gene that could provide valuable reference ideas for the treatment of PD. In a zebrafish PD model deficient *DJ-1*, p53 and the proapoptotic factor Bax were overexpressed before toxin exposure, and no significant neuronal cell death was observed, suggesting that subliminal activation of the cell death pathway may have therapeutic effects on PD, such as inhibition of the p53 pathway (Bretaud et al., 2007). p53 activation can also aggravate the oxidative damage of dopaminergic neurons by downregulating the expression of proliferating cell nuclear antigen in a PD cell model (Li et al., 2016). Similarly, a PD cell model was induced by the l-methyl-4-phenyl-l,2,3,6-tetrahydropyridine neurotoxin, which is an inducer of chronic and irreversible PD. p53 mediates oxidative damage by downregulating proliferating cell nuclear antigen and further promotes programmed neuronal death, which is reasonably believed to partly promote the degeneration and shrinkage of dopaminergic neurons in the brain (Qi et al., 2016).

p53 can regulate the functional activity of HSP70 chaperones in neurodegenerative diseases. Studies in the PD cellular model SH-SYSY and PD patients have shown that activation of p53 can inhibit the protein folding involved in hsp70 and increase the collection of α-synaptonucleoproteins in neurons (Chen et al., 2020). Parkin is a neuroprotective protein with a transcriptional inhibitory function of p53, and its mutation is closely related to the pathogenesis of PD (Luo et al., 2022). Parkin-mediated mitophagy is important for clearing damaged mitochondria and maintaining the quality of mitochondria, but p53 activation can disrupt this state under pathogenic conditions (Goiran et al., 2018). In the normal physiological state, parkin can interact with p53 and then inhibit p53 transcription. Meanwhile, p53 regulates the transcription of parkin to keep it at a relatively stable level (Checler and Alves da Costa, 2014). However, when parkin is mutated in PD patients, the transcriptional function to repress p53 is abolished, and overexpression of p53 reduces or even inactivates the autophagy activity of parkin, resulting in impaired autophagy function and neurodegeneration (Huang et al., 2021). Therefore, p53-dependent therapeutic intervention may provide potential targets for novel neuroprotective drugs for PD.

## P53 AND AUTISM

Autism spectrum disorder (ASD) is also known as autism. Its clinical manifestations include social interaction difficulties, communication impairment, and stereotyped repetitive behavior patterns, which are the result of a combination of genetics and external environmental factors (McPartland and Volkmar, 2012). However, ASD susceptibility genes and their functions are largely unknown. Studies have shown that mitochondrial dysfunction and abnormal expression of mitochondrial DNA were significantly higher in autistic children than in controls (Giulivi et al., 2010). In the peripheral blood mononuclear cells of children with autism, researchers have detected the deletion of mitochondrial DNA and high expression of the p53 gene (Wong et al., 2016). In the ASD rat model with arsenic exposure, the serum levels of p53 were higher than in healthy controls, as well as the changes in ASD children (Zhou et al., 2020). Therefore, the higher expression of p53 involved in the pathogenesis of ASD resulted from environmental factor–induced brain mitochondrial dysfunction. In addition, activation of p53-dependent apoptosis also impeded the growth of the embryonic cerebral cortex in an ASD mouse model with deletion of CRM1, a gene commonly deleted in ASD patients (X. Li et al., 2020). Chromodomain helicase DNA-binding protein 8 (CHD8) is mutated in individuals with autism spectrum disorder, and heterozygous mice with CHD8 mutations are known to exhibit significant ASD properties (Katayama et al., 2016; Nita et al., 2021). Abnormal expression of CHD8 can regulate p53-dependent apoptosis genes, cell cycle arrest, and neural progenitor cell self-renewal, which are involved in the occurrence of ASD (Xiong et al., 2020). The posttranslational modification of p53 is involved in regulating neuron dendrite growth and branching. Repression of p53 acetylation was detected in another ASD mouse model with ANKRD11 gene deletion or truncation mutation, which regulates pyramidal neuron migration and dendritic differentiation in the developing cerebral cortex (Ka and Kim, 2018). According to reports, p53 also regulates some microRNAs involved in the development of ASD, including miR-19b-1, miR-34c, miR-15a, and miR-23b (Bourgeron, 2009; Zhang et al., 2016; Huang et al., 2021).

## P53 AND SPINOCEREBELLAR ATAXIA (SCA)

SCA is an autosomal dominant genetic disease that mainly manifests as behavioral movement disorders and speech disorders. There is currently no specific drug treatment, so it is very important to study its pathogenesis and pathways (Niewiadomska-Cimicka and Trottier, 2019). The pathogenic mechanisms of SCA are caused by polyglutamine ataxia, which belongs to the polyglutamine family type of neurodegenerative disease (Wang et al., 2010; Ajayi et al., 2015). Spinocerebellar ataxia type 3 (SCA3) is a Machado-Joseph disease. This is one of the relatively common types of inherited ataxia. In cell and animal models of SCA3 induced by ataxin-3 mutation, mutant SCA3 can induce apoptosis of cerebellum and pons neurons by enhancing p53 transcriptional activity (Chou et al., 2011). Similarly, in the animal model of SCA7, which is caused by ataxin-7 mutation, the same conclusion was obtained. Interestingly, when disrupting p53 function in an SCA7 cell model, cellular metabolic dysfunction could be reversed (Ajayi et al., 2015). Moreover, enhanced phosphorylation of p53 and the activity of transcription factors contributed to SCA7 regulation of cerebellar and inferior olivary nucleus neuronal death and were accompanied by mitochondrial respiratory chain damage in SCA7 disease (Wang et al., 2010). Therefore, the inhibition of aberrant p53 activation, such as the p53 inhibitor pifithrin-a, might provide a potential target for the clinical treatment of these polyglutaminergic nerve diseases.

## P53 AND OTHER CNS DISEASES

p53 is not only involved in the pathogenesis of brain tumors, AD, and spinocerebellar ataxia, but also in the occurrence and development of other CNS diseases, such as schizophrenia (SCZ), depression, epilepsy, and cerebral ischemia. SCZ and depression are common affective disorders with a high incidence (Upthegrove et al., 2017). It has been shown that increased apoptosis of neurons may account for neurodevelopmental abnormalities as well as SCZ, so p53 might be a candidate risk gene in SCZ (Catts and Catts, 2000). To test the association between p53 and SCZ, the researchers analyzed 286 SCZ cases and 264 controls, and the results confirmed a significant association between TP53 and SCZ (Ni et al., 2005). However, the authors also showed that the p53 polymorphisms rs1042522 and rs17879353 contributed to susceptibility to bipolar disorder but not to SCZ in the Chinese Han population (Yang et al., 2019). In a mouse model of bilateral ovariectomy, when mice had fewer neurons in areas of the prefrontal cortex, hippocampus, or amygdala, these mice exhibited depression-like and dementia-like behavior. The detection data showed strong phosphorylation of p53 (Fang et al., 2018). Similarly, in a sleep disorder mouse model, bioinformatics analysis showed significant activation of the p53 signaling pathway. In addition, p53 protein-activated death domain protein 1 is an important gene involved in the pathogenesis of depression and exercise intervention diseases. These findings also provide new ideas for the treatment of clinical depression (Liu et al., 2022). Depression and tumors often occur together, and p53 also plays a very important role in tumorigenesis, so it is worth considering and of great interest to explore whether we can simultaneously treat comorbidities by regulating p53.

Epilepsy is a common neurological disease with a complex etiology. In addition, p53 is significantly upregulated in both posttraumatic epilepsy and drug-resistant epilepsy rat models, accompanied by increased apoptosis of nerve cells in the mesial temporal lobe and hippocampus (Huang et al., 2020; Wang et al., 2021a; Wang et al., 2021b). Similarly, the occurrence of cerebral ischemia and cerebral infarction is closely related to apoptosis or cell death processes (Qin et al., 2022). In the rat transient global cerebral ischemia model, apoptosis of hippocampal CA1 neurons is closely related to p53 translocation to mitochondrial accumulation, and p53 also induces the activation of releasing cytochrome c in mitochondria (Endo et al., 2006). Another study found that pifithrin-α protected neurons in certain neurological diseases. In the cerebral ischemia model, the infarct size in the cortical area was greatly reduced after treatment with PFT. It was speculated that PTF affects the translocation of p53 to the nucleus, thereby exerting an inhibitory effect on p53 and thus reducing apoptosis (Leker et al., 2004). Further research found that high doses of PFT can inhibit the expression of PUMA and NOXA, while low and moderate doses also inhibit p53 accumulation in mitochondria (Endo et al., 2006). In addition to PFT, the traditional Chinese medicine Xiao-Xu-Ming decoction could also significantly improve the apoptosis damage caused by cerebral ischemia. It not only reduces the expression of p53 and Bax but also increases the expression of Bcl2 in mitochondria (Lan et al., 2014). In general, the development of drugs targeting p53 to inhibit apoptosis in cerebral ischemic diseases can greatly alleviate the symptoms of patients and provide a reference for clinical practice.

## CONCLUSIONS

**Figure 2.**
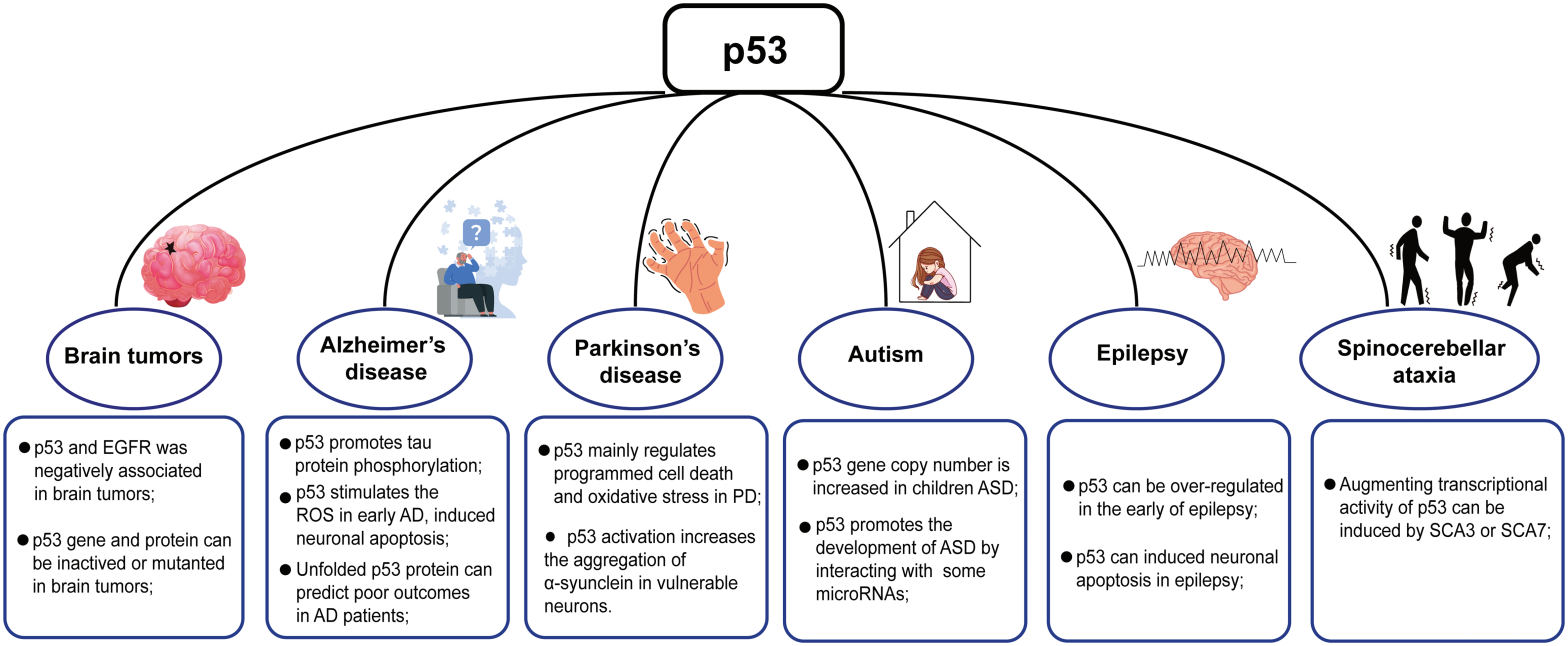
The contribution of p53 in central nervous system (CNS) diseases. The diagram was drawn and generated with the help of Figdraw (www.figdraw.com).

In this review, we describe the role of the p53 pathway and genetic alterations of p53 in the progression of different CNS diseases (**Figure 2**). At present, evidence for the role of p53 in related neurological diseases is still relatively limited and needs more research support. In addition, as people grow older, the risk of co-occurrence tumor and neurodegenerative diseases increases significantly; whether and how p53 is involved in this process should be given more attention. It should be noted that most preclinical models are constructed using adult animals, which ignores the effect of aging and may hinder hypothesis clarification (Li et al., 2019a). Therefore, aged animals should be given priority to avoid these issues in the future. Furthermore, the detection of p53 and its related signaling pathways is mostly carried out on tumor tissues, which is not applicable to human brain tissues due to logistical and ethical obstacles. Thus, alternative samples such as cerebrospinal fluid and peripheral blood have been widely applied in neurological diseases. To better understand the status of p53 in the brain, it is essential to identify new biomarkers and detection methods for p53 in these proxy samples, which can pave the way for future research.

Studies have demonstrated a strong correlation between tumors, neurological disorders, and p53. Consequently, antitumor drugs that target p53 may have potential therapeutic benefits for treating AD, PD, autism, and so on. Some of these drugs function by inhibiting the ubiquitination degradation of p53, which is regulated by E3 ligase MDM2 (H. Wang et al., 2023). To restore the normal function of wild-type p53, drugs such as Nutlins and Spirooxindoles can work through blocking the binding of MDM2-p53, downregulating MDM2 expression, or inhibiting its activity (Marvalim et al., 2023). Additionally, certain drugs including Prima-1, APR-246, PhiKan083It, ReACp53, ADH-6, and L^I^/L^H^ have been utilized to reverse the abnormal structure of p53 or downstream signaling pathway changes caused by p53 mutation (Boeckler et al., 2008; Wiman, 2010; Soragni et al., 2016; Miller et al., 2019; Palanikumar et al., 2021; H. Wang et al., 2023). Some compounds have shown promise in unfolding p53 aggregation or targeting specific mutations, such as Y220C mutation. So, in the future, more in vitro and in vivo experiments are needed to fully assess the therapeutic effects of these drugs on neurological diseases, which may shed light on the treatment of some intractable neurological diseases.

In addition, when discussing p53, the role and function of its family members p73 and p63 should not be forgotten (Agostini et al., 2018). According to reports, p73 is widely expressed in the nervous system, and p73 collaborates with p53 to participate in the regulation of neural development and the pathogenesis of some neurological diseases (Nemajerova et al., 2018). Thus, p53 protein may regulate the survival, development, and differentiation of neurons in the brain through interactions with family members (Levrero et al., 2000). Whether the interaction relationship between the p53 protein family can be used to treat or prevent neurological diseases is a question worth considering to provide a comprehensive understanding of the molecular mechanism(s) involved in the p53 family, which could extend our understanding of the occurrence and progression of central nervous system diseases and provide ideas for new therapeutic approaches.
